# Atomistic electrodynamics simulations of bare and ligand-coated nanoparticles in the quantum size regime

**DOI:** 10.1038/ncomms9921

**Published:** 2015-11-10

**Authors:** Xing Chen, Justin E. Moore, Meserret Zekarias, Lasse Jensen

**Affiliations:** 1 Department of Chemistry, The Pennsylvania State University, 104 Chemistry Building, University Park, Pennsylvania 16802, USA

## Abstract

The optical properties of metallic nanoparticles with nanometre dimensions exhibit features that cannot be described by classical electrodynamics. In this quantum size regime, the near-field properties are significantly modified and depend strongly on the geometric arrangements. However, simulating realistically sized systems while retaining the atomistic description remains computationally intractable for fully quantum mechanical approaches. Here we introduce an atomistic electrodynamics model where the traditional description of nanoparticles in terms of a macroscopic homogenous dielectric constant is replaced by an atomic representation with dielectric properties that depend on the local chemical environment. This model provides a unified description of bare and ligand-coated nanoparticles, as well as strongly interacting nanoparticle dimer systems. The non-local screening owing to an inhomogeneous ligand layer is shown to drastically modify the near-field properties. This will be important to consider in optimization of plasmonic nanostructures for near-field spectroscopy and sensing applications.

## Abstract (web summary)

Investigating the properties of metallic nanoparticles in the 2–10 nm range is a computational challenge. Here, the authors introduce an atomistic electrodynamics model to describe bare and coated particles and dimers, and show that the ligand layer modifies the near-field properties of the particles.

## Graphical abstract

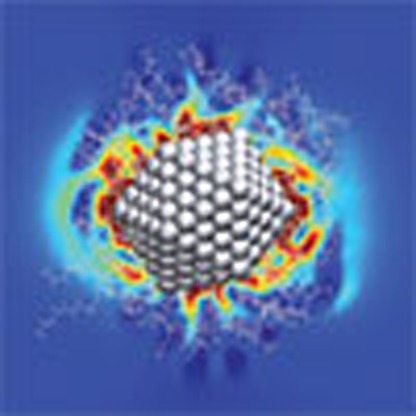

The ability to localize and manipulate light at the nanoscale well below the diffraction limit makes plasmonic nanoparticles unique. Hence, plasmonic nanoparticles offer advantages in a large array of applications such as energy harvesting^1^, plasmon-assisted chemistry^2^,^3^, chemical sensing^4^ and using the plasmonic near-field to enhance molecular spectroscopy^5^. While classical electrodynamics simulations are typically used to explain the optical properties of the plasmonic nanoparticles, recent works^6^,^7^,^8^,^9^,^10^ have clearly demonstrated that this description fails for small nanoparticles and aggregates with small separations.

For individual nanoparticles in this quantum size regime (diameter<10 nm), a significant blue shift of the plasmon resonance is observed in contrast to classical predictions^7^,^11^,^12^. This blue shift arises from the smearing of the electronic charge distribution over the particle surface and is accounted for both in quantum mechanical^13^ and nonlocal electrodynamics^14^ models. Nanoparticle dimers with separations less than their radius are of particular interest owing to a plasmon shift and a significantly enhanced electromagnetic field in the junction arising from strong coupling. The large field is utilized in surface-enhanced spectroscopy. For nanoparticle dimers, the quantum size regime occurs when the separation becomes less than ∼1 nm (ref. 6). At these small distances the electronic charge distribution between the two nanoparticles overlaps, leading to a reduction of the field in the junction and a smooth transition between touching and non-touching particles^10^. This puts a fundamental limit on how large the field enhancements in the junction can be^8^,^9^,^15^,^16^. These effects are correctly described using time-dependent density-functional theory (TDDFT) simulations and are interpreted as the effect of electron tunnelling between the two particles. A recently developed quantum-corrected electrodynamics (QCE) model that incorporates the gap tunnelling into classical simulations has been shown excellent agreement with the TDDFT simulations^10^. The generalized nonlocal optical response (GNOR) model correctly produces the smooth transition in the contact region by accounting for the electron diffusion at the nanoparticle’s surface^14^.

Much effort has been focused on understanding the quantum size effects of bare particles. However, recent work has shown that molecules attached to the surface significantly alter the plasmon in the quantum regime. A reversal of the expected blue shift was observed for small ligand-covered particles^17^. Furthermore, it has been shown that molecules placed in the junction between two nanoparticles can tune the plasmon by modulating the tunnelling between the particles^18^. Clearly it is important to understand the optical response of the particles in the quantum regime accounting for the interactions with the ligands. Quantum mechanical simulations can describe ligand-covered particles as large as 2 nm (sufficiently large enough to support plasmon excitations), but remain computationally intractable for larger systems^19^. Both the QCE and GNOR models treat the ligand layer as a homogeneous environment described by a local dielectric constant and thus cannot account for the specific interactions with the metal surface.

In this work we introduce an atomistic electrodynamics model for the simulations of bare and ligand-coated nanoparticles in the quantum size regime that naturally incorporates the effects of the local chemical environment through atomic coordination dependence. In this coordination-dependent discrete interaction model (cd-DIM), the nanoparticle is described by a collection of atoms that are assigned an atomic polarizability representing a spherical charge distribution with properties that depend on its local environment. Thus, cd-DIM can be considered an atomistic variant of the popular discrete dipole approximation (DDA) which is commonly used to simulate plasmonic properties of large metallic nanoparticles^20^,^21^. Using cd-DIM, we present a unified description of both bare and ligand-coated nanoparticles in the quantum size regime. We show that this model correctly describes the strongly interacting nanoparticle dimer system, including the reduction of the near-field in the junction, in good agreement with recent quantum mechanical simulations. This opens up the ability to perform atomistic simulations of the optical properties of realistic inhomogeneous plasmonic systems. This will be important for further optimization of plasmonic nanostructures for near-field spectroscopy and sensing applications.

## Results

### The coordination-dependent discrete interaction model

**Figure 1:**
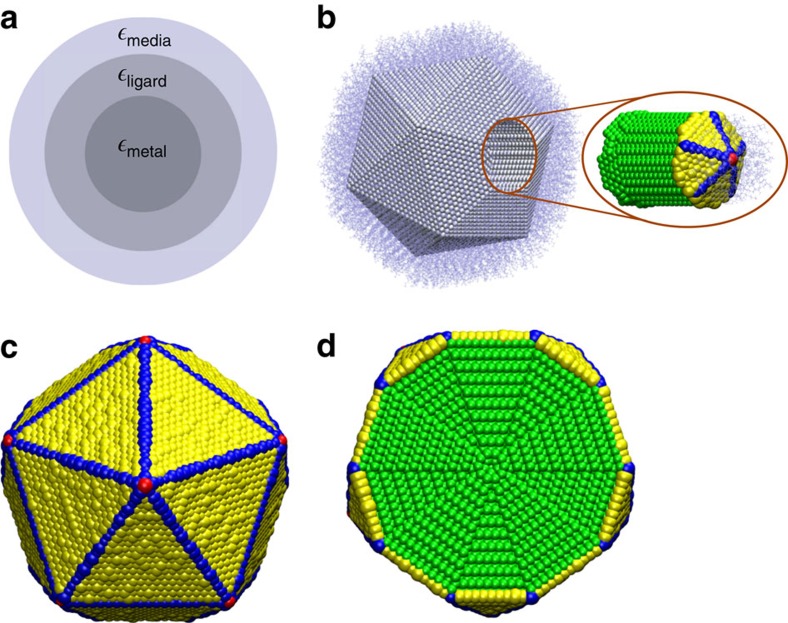
Computational models for coated Ag nanoparticles. The traditional continuum model is presented in **a** and contrasted with the atomistic representation of Ag nanoparticles coated by ligands in **b**. The local environment of the nanoparticle surface is shown in **c** together with a cross-section view in **d**. The atomic spheres are colour coded according to their local coordination number, where red indicates low coordination numbers and green indicates the coordination number for bulk Ag. The atomic radii are scaled by the coordination number of Ag binding to the nitrogen atoms of the ligands.

The central idea of cd-DIM is illustrated in Fig. 1, where the traditional description of nanoparticles in terms of a macroscopic homogenous dielectric constant is replaced by an atomic representation with dielectric properties that depend on the local chemical environment. In this way, we can differentiate between surface and bulk atoms as well as local chemical interactions with the ligands, here oleylamine (OAm), which binds through the nitrogen atom. In cd-DIM, the total energy of a set of interacting fluctuating dipoles is given by


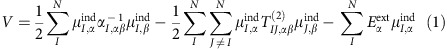


where the first term is ascribed to the self-energy, the second term is resposible for the dipole–dipole interaction energy and the last term arises from the interactions with the external field. Here *α*_*I*,*αβ*_ represents a component of the atomic polarizability tensors of atom *I* and 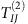 is the second-order interaction tensor describing the interactions between dipole *I* and *J*. In cd-DIM, Gaussian charge distribution is used to describe the polarizable sphere. The interaction tensor is therefore renormalized which effectively screens the interactions at short distances^22^,^23^. In contrast, the DDA model treats the atoms as point objects and thus the bare unscreened interaction tensor is used. Retardation effects are not included because of the small size of the nanoparticles studied; however, this could be straightforwardly included using the fully retarded interactions tensor as is common in the DDA method^20^,^21^. The total polarizability can then be found by minimizing the total energy with respect to the induced atomic dipoles which leads to the following set of linear response equations


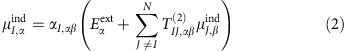


which are solved self-consistently using an iterative solver combined with a fast multilevel cell—multipole matrix—vector multiplication scheme^24^. The total polarizability is then found as


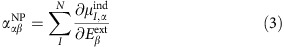


from which the optical absorption across section at a frequency *ω* can be obtained as


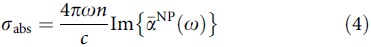


where *n* is the refractive index of surrounding medium and 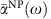 is the isotropic polarizability of the whole system. The electric field around the nanoparticle can be obtained directly from the atomic dipoles using either the renormalized interaction tensor with a probe radius of 1.58 Å for cd-DIM or the bare interaction tensor for DDA. The atomic polarizability is taken to be isotropic and obtained from a Clausius–Mossotti relation as


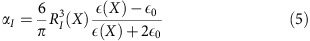


where *R*_*I*_(*X*) is the coordination-dependent radii of the atom, *ϵ*(*X*) is the coordination-dependent dielectric constant of the material and *ϵ*_0_ is the dielectric constant of the environment. The specific parametrization of the coordination-dependent polarizability and dielectric constant is given in the Methods section.

### Plasmonic response of bare Ag nanoparticles

Quantifying the optical properties of metallic nanoparticles with diameters <10 nm is difficult because of the strong dependence on size, shape and local environment for such small nanoparticles. Recent works on bare nanoparticles using scanning transmission electron microscopy electron energy-loss spectroscopy have demonstrated a 0.5 eV blue shift of the plasmon excitation as the size of the nanoparticle decreases from 10 to 2 nm (refs 7, 12). This blue shift is consistent with that found in small, molecular-like Ag clusters^11^. Simulations based on quantum mechanical^25^ and nonlocal electrodynamics^26^ models have shown that the origin of this blue shift can be ascribed to a smearing of the charge density at the surface with the charge centroid pushed towards the particle centre due to the screening from the bound d-electrons^26^.

**Figure 2:**
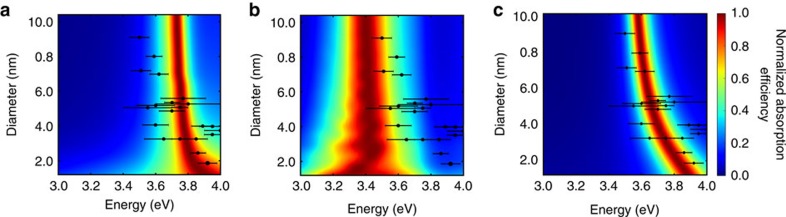
Optical absorption of bare Ag nanoparticles with different diameters. Comparison between theoretical predictions and experimental EELS measurements. The calculations are carried out by using (**a**) the cd-DIM, (**b**) the DDA model and (**c**) the GNOR model, respectively. The experimental data is taken from ref. 7, where the horizontal error bars indicate 95% confidence intervals. To directly compare with the simulated spectra the experiment spectrum was blue-shifted by 0.2 eV to remove solvent effects. In all the spectra the absorption efficiency has been normalized for each nanoparticle.

By differentiating between surface and bulk atoms, we show that cd-DIM correctly describes the blue shift of the plasmon excitation as the size decreases. In Fig. 2a, the absorption spectra of icosahedral nanoparticles simulated using cd-DIM are compared with the experimental data obtained for small spherical particles using electron energy-loss spectroscopy^7^. Good agreement with the experimental results is found, especially for diameters <6 nm. For the larger diameters, we see that the plasmon excitation is slightly larger than the experimental results. The observed blue shift is the result of a combination of spill-out effects and quantum size effects incorporated into the coordination-dependent dielectric constants. In cd-DIM, each atom is described by a spherical charge distribution with a radius that depends on the local coordination environment. Atoms at the surface are assigned a larger radius than atoms in the bulk to account for spill-out effects at the surface. The dipole–dipole interactions between the atoms are renormalized owing to the smearing of the charge distribution which leads to a blue shift of the plasmon excitation. Furthermore, in cd-DIM the surface atoms are also described by a modified dielectric constant, where the plasmon frequency is pushed to higher energies to reflect the less free-electron behaviour at the surface. This also contributes to a blue shift of the plasmon excitation. In contrast, when the atoms are treated as point-like objects described by a local bulk dielectric constant, the expected size-independent plasmon excitation is found as illustrated in Fig. 2b. For comparison, results from the quasi-static limit of the GNOR model which incorporated nonlocal effects are shown in Fig. 2c which are in good agreement with the experimental data over the whole size range. Both the results from the GNOR model and cd-DIM find a somewhat smaller blue shift than experiments find. Substrate effects have been ruled out as the origin for this discrepancy^27^. Finally, the cd-DIM results for the larger nanoparticles are a little higher in energy than both the GNOR results and what is expected from classical electrodynamics. This is likely because of the parametrization of the radii in terms of the static polarizabilities against TDDFT data for small metal clusters, see Supplementary Fig. 1 and Supplementary Methods for details.

### Plasmonic response of bare Ag nanoparticle dimers

**Figure 3:**
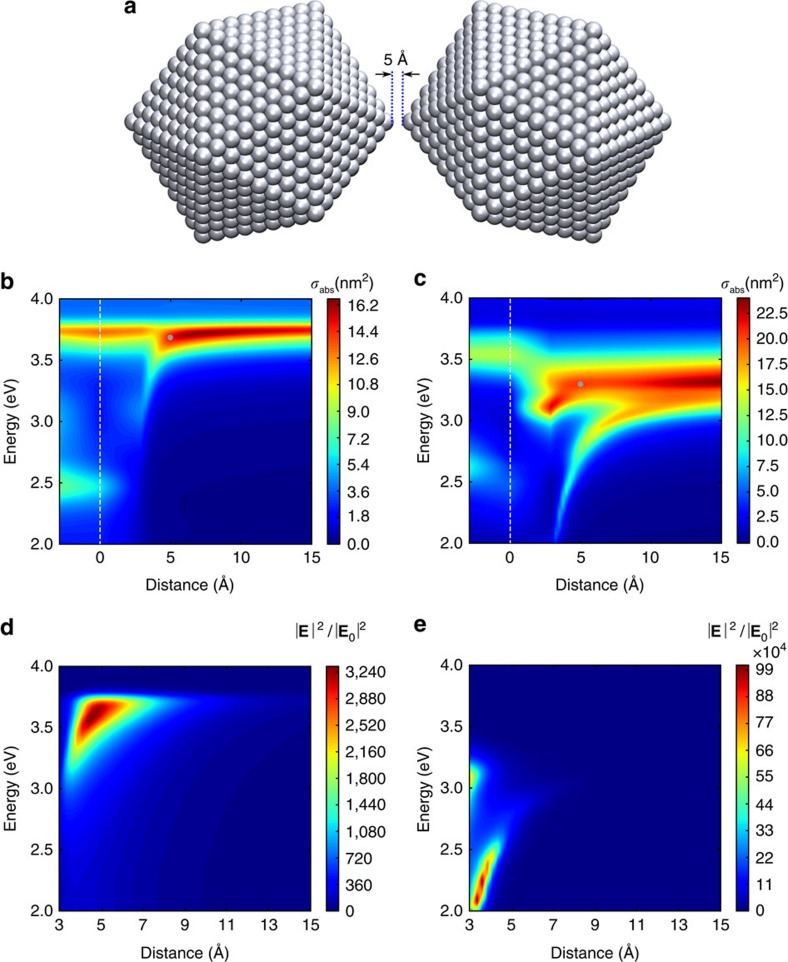
Optical properties of a dimer of two icosahedral Ag nanoparticles. The two Ag icosahedral nanoparticles are aligned with the fivefold axes of rotation and the gap is defined as the length between two tip Ag atoms (**a**). Both the absorption cross-section (in nm^2^) and the electric field enhancement 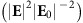 are calculated by cd-DIM (**b**,**d**) and DDA (**c**,**e**) aligned with the incident field polarized along the dimer axis. The white dashed line denotes the gap distance of zero and the grey dot represents the dimer with a separation distance of 5 Å.

Two strongly coupled plasmonic dimers separated by a small distance is another example where classical electrodynamics is known to fail in describing the optical properties. Here we show that cd-DIM correctly describes the physics of this system even for very short separations owing to the inclusion of non-local screening. The absorption spectra of two icosahedral Ag nanoparticles with a diameter of 4.6 nm are shown in Fig. 3. The two nanoparticles are oriented such that their vertices are aligned along the axis of separation, and the distance refers to the separation between the two Ag vertex atoms, as demonstrated in Fig. 3a. For separations >5 Å, we are in the non-contact regime where classical electrodynamics is valid, whereas for distances smaller than that we are in the bonding regime. For zero separation, the nanoparticles share a common vertex atom and start to form a single unified nanoparticle for which a classical description again becomes valid. It is in the intermediate regime where nonclassical behaviour is observed and where we see the largest difference between the cd-DIM and DDA results. The results obtained using cd-DIM show a gradual red-shifting and weakening of the main dipolar plasmon around 3.7 eV, a smooth transition between the quadrupolar plasmon and the high-energy charge-transfer plasmon at short distances, and the emergence of a low-energy charge-transfer plasmon around 2.5 eV. This result is in good agreement with the recent TDDFT, QCE and GNOR predictions. In contrast to this, the DDA results show a more discontinuous transition in the bonding regime with the lowest charge-transfer plasmon only emerging when the nanoparticles overlap significantly. In cd-DIM the emergence of the charge-transfer plasmon in the contact region is captured through the coordination dependence of the atomic polarizabilities. As the distance between the two nanoparticles in the dimer gets smaller the nature of the vertex atom changes continuously from being surface-like to being bulk-like. This change in character of the atom leads to both a change in the spill-out effect through a change in the atomic radii and a change in its dielectric constant to make it more free-electron like. These effects combined is what leads to this improved description at small distances and the correct description of the charge-transfer plasmon.

In addition, the magnitude of the electric field enhancement in the centre of the gap depends strongly on the separation as shown in Fig. 3d,e for cd-DIM and DDA, respectively. For cd-DIM, the largest field enhancements are found around 5 Å in good agreement with predictions from TDDFT and QCE. The field in the junction is completely screened for the shortest separations due to the smearing of the charge distribution in the cd-DIM model. The results obtained using DDA show a completely different picture with the strongest enhancements at the shortest distances. Furthermore, in DDA the enhancements track the dipole plasmon leading to the largest fields around 2 eV, and the enhancements are much larger than those predicted from cd-DIM. While the GNOR model correctly predicts a screening of the electromagnetic field in the junction, the field enhancements still track the dipolar plasmon and becomes the strongest at the shortest distances, see Supplementary Fig. 2. This is owing to the neglect of the overlap of the spill-out charge distribution in the GNOR model which becomes important for distance below 5 Å (ref. 28). Overall, the cd-DIM succeeds in describing the optical properties of strongly coupled metallic dimers with sub-nanometre gaps in good agreement with the accurate predictions based on TDDFT.

### Plasmonic response of ligand-coated Ag nanoparticles

In contrast to the large amount of simulations carried out for bare nanoparticles, much less has been done for the ligand-coated nanoparticles. The ligand layer is typically described as a homogenous layer with a small real refractive index. However, recent work has shown that a OAm ligand layer can cause a reversal of the blue shift in the small bare Ag nanoparticles^17^. This reversal was attributed to a reduction in the conductivity of the outer layer of metal atoms caused by the binding ligands. Simulations based on a multilayer Mie theory where the outer layer was described using a modified dielectric constant with reduced conductivity were shown to agree with the experiments. So far simulations that provide a realistic description of the ligand layer have not been pursued beyond TDDFT simulations of small monolayer-protected metal clusters^19^.

**Figure 4:**
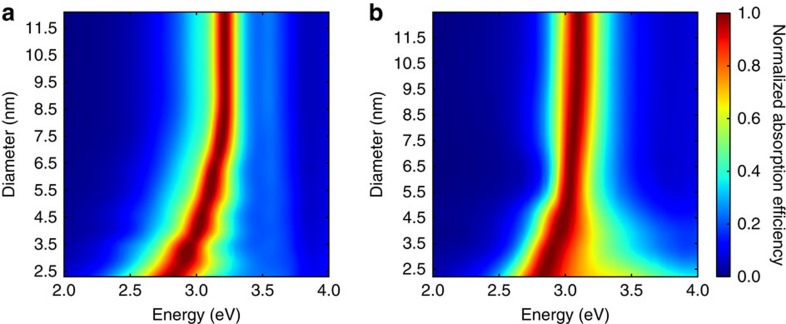
Optical absorption cross-section of ligand-coated Ag nanoparticles. The comparison between (**a**) the simulation results generated by cd-DIM and (**b**) the experimental results taken from ref. 17. Ag nanoparticles with diameters ranging from 2.3 to 12.1 nm are considered. The absorption efficiency is normalized for each nanoparticle and the surrounding medium is described by a refractive index of *n*=1.379.

In cd-DIM the interactions between the ligands and Ag atoms on the surface of the metallic nanoparticles cause a local modification of the atomic polarizability which leads to the red-shifting of the plasmon response for small nanoparticles. The interactions with the ligands cause a reduced conductivity due to the formation of the chemical bond^17^. This is illustrated in Fig. 4a where we plot the plasmonic absorption spectra of various coated Ag nanoparticles with diameters ranging from 2.3 to 12.1 nm obtained from cd-DIM calculations. For comparison, the experimental results are shown in Fig. 4b. Overall we see good agreement between the cd-DIM results and the experimental observations in spite of a few slight differences. The simulations predict a 0.42 eV shift, whereas a slightly smaller shift of 0.3 eV is found in the experiments. Also, we see that the experimental spectra are broader compared with the simulations especially for the smaller particles, see Supplementary Fig. 3. This is likely caused by experimental deviations from a perfect icosahedral geometry. We can eliminate the discrepancy by applying a slightly increased size-dependent damping parameter, however, we did not choose this to preserve the weaker bands on either side of the main peak due to the perfect icosahedral structure used in the simulation.

**Figure 5:**
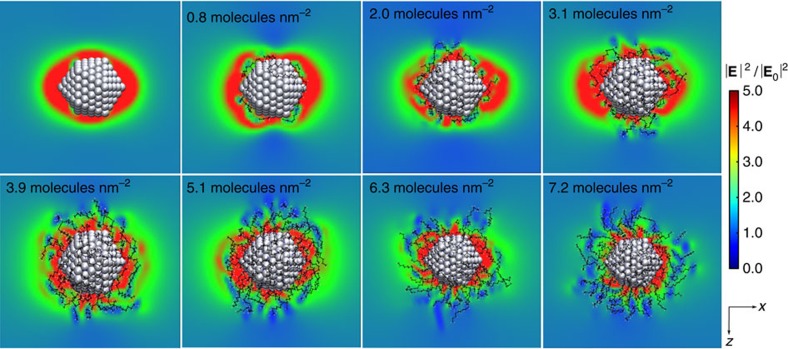
Electric field distribution around coated Ag nanoparticle. The electric field distribution is obtained with the incident field polarized along the long axis. A refractive index of *n*=1.379 is used for the surrounding medium. Changing the coverage from 0 to 7.2 molecules per nm^2^ shows the evolution of the electric field distribution as a monolayer is formed.

The main advantage of the cd-DIM model is that it retains the atomistic description of the nanoparticle and the ligand layer. This allows us to understand how the ligand layer perturbs the electric field around the nanoparticle under the nonlocal screening effects. Typically the ligand layer is treated as a homogenous medium described by a local dielectric constant where the inhomogeneous nature of the ligand layer and the nonlocal screening have not been considered. Here we examined this by considering the electric field around a small nanoparticle as a function of the ligand coverage ranging from 0.8 to 7.2 molecules per nm^2^. The results in Fig. 5 clearly show that as the ligand coverage increases, the local field around the nanoparticle is screened in the vicinity of the ligands. Once a monolayer of ligands is formed, the field is significantly perturbed and looks nothing like the initial field around the bare particle. Since the nanoparticle is small, the dimensions of the ligand layer are comparable with the nanoparticle’s size. This causes the ligand layer to be highly disordered which is then reflected in the field distribution around the nanoparticle.

**Figure 6:**
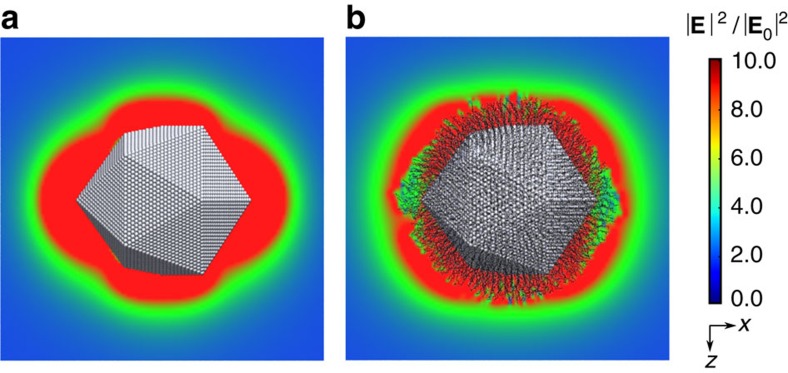
Electric field distribution around bare and coated Ag nanoparticles. (**a**) Bare Ag nanoparticle and (**b**) coated Ag nanoparticle at the incident field polarized along the long axis. In the simulations the surrounding medium is described with a refractive index of *n*=1.379.

For larger nanoparticles one could envision that the field would be less perturbed by the presence of the ligand layer due to the larger relative dimensions of the nanoparticle and that the ligand layer is more homogeneous. In Fig. 6 we examined this by considering the near-field around a bare and ligand-coated icosahedral nanoparticle with a diameter of 12.1 nm with the incident electric field polarized along the long axis. Even though the near-field now extends beyond the ligand layer, it is still significantly modified in the vicinity of the surface. It is especially noteworthy that the near-field is significantly decreased around the nanoparticle tips. Consequently, the regularity of the electric field enhancement found for bare icosahedral Ag nanoparticle is not preserved in coated nanoparticle which is likely to affect the near-field coupling between nanoparticles that are used in sensing applications.

## Discussion

In summary, we have presented an atomistic electrodynamics model that provides a unified description of bare and ligand-coated nanoparticles, as well as strongly interacting nanoparticle dimer system in the quantum size regime where traditional classical models fail. We have shown that the model describes the blue shift of the plasmon in small Ag nanoparticles, the reversal of this effect due to interactions with ligands, and the reduction of the near-field in strongly coupled nanoparticle dimer system. Furthermore, we have demonstrated that non-local screening due to an inhomogeneous ligand layer around the nanoparticle leads to a significant modification of the near-field. This will be important to consider in applications that rely on the strong interactions between nanoparticles coated by inhomogeneous ligand layers such as plasmonic rulers^29^ and the recent demonstration of molecular tuning of quantum plasmon resonances^18^,^30^. Furthermore, we expect that the atomistic description combined with the non-local screening will be necessary for understanding surface-enhanced spectroscopies as they push towards sub-nm resolution^31^.

## Methods

### Paremeterization of cd-DIM

To account for the different environments of the Ag atoms in the nanoparticle (that is, surface, edge, vertex and core), the effective coordination-dependent radius is introduced^32^


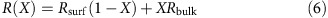


where


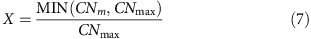


where *CN*_max_ is the maximum coordination numbers for the atom type (*CN*_max_=12 for Ag), and *CN*_*m*_ is the effective coordination number for DIM atom *m*. The effective coordination number is calculated from^32^


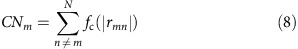


where the cutoff function is given by


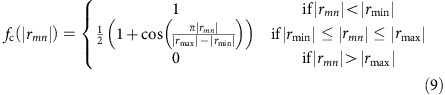


which smoothly connects atoms inside and outside of the coordination sphere. The advantage of using an effective coordination number compared with a standard coordination number (that is, integer value) is that the effective coordination number can account for defects in the nanoparticle, or the static case we use *ϵ*=∞ and thus the polarizability reduces to the volume of the atom as expected for a perfect conductor. The following atomic radii were used, *R*_Ag,bulk_=1.56 Å, *R*_Ag,surf_=1.65 Å, *R*_C_=0.82 Å, *R*_N_=0.76 Å and *R*_H_=0.63 Å. They were obtained by parameterizing the static polarizability as described in the Supplementary Method and Supplementary Fig. 4.

Within the cd-DIM framework, the coordination-dependent dielectric constant is represented as


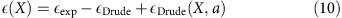


where *ϵ*_exp_ is taken from Johnson and Christy^33^. In equation 10, the Drude function is given by


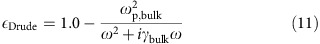


and


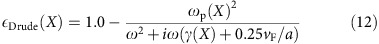


where *a* is the radius of the particle, and


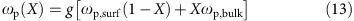


with *ω*_p,surf_=22.0 eV, and *ω*_p,bulk_=9.6 eV. The *g* factor accounts for the lowering of the optical conductivity owing to the interactions with the ligand layer^17^ and is given by


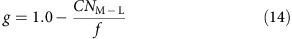


where *CN*_M−L_ represents the coordination number of metal atoms binding to the atoms in the ligands. In this work, the parameter *f* is given as 0.7, leading to *g*∼0.8 for a monolayer coverage in agreement with the previous work based on a multilayer Mie model^17^. A size-dependent damping correction is adopted where *ν*_F_ represents the Fermi velocity and


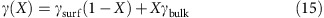


with *γ*_surf_=2.0 eV and *γ*_bulk_=0.0228, eV.

### GNOR simulations

The non-local polarizabilities for spheres of various radii were calculated within the quasi-static limit of the GNOR model as^14^


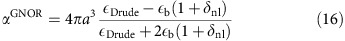


where the non-local correction factor is given by


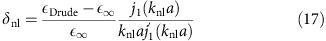


with the non-local factor given by


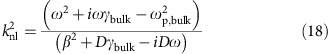


where the hydrodynamic parameter is given by 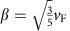, and the diffusion parameter *D* was fitted such that the full width at half maximum (FWHM) of a single particle using GNOR matched the FWHM using the local-response approximation with a size-dependent Drude damping parameter^34^. The bound dielectric term (*ε*_∞_) was described using a frequency-dependent polynomial form found in ref. 12 but rescaled by 0.948 to better match the Johnson and Christy^33^ dielectric for Ag. The background dielectric (*ε*_b_) was 1.0, and the Fermi velocity of Ag (*v*_F_) was 1.39 × 10^6^ m s^−1^ (ref. 35).

### Molecular dynamics simulations

We performed molecular dynamics (MD) simulations using the Large-scale Atomic/molecular Massively Parallel Simulator (LAMMPS, http://lammps.sandia.gov.)^36^ to obtain the structures of the Ag nanoparticle coated by the OAm ligands. The initial structure of the OAm ligands were chosen to be perpendicular to the Ag surface forming a compact monolayer, and then the coated nanoparticle was immersed into a cubic box filled with OAm molecules. The initial surface coverage was set to be 5.5 molecules per nm^2^ (ref. 37). For the MD force-field we used the General AMBER force field^38^,^39^ to describe the intramolecular interactions of OAm ligands. To describe the interactions between the ligands and the nanoparticle we adopted a modified Ag-OAm embedded-atom-method potential as described in the Supplementary Methods, Supplementary Figs 5–6 and Supplementary Table 1. After the energy minimization, the whole system was slowly heated up and equilibrated at 100 K in order to yield a condensed ligand zone around the Ag cluster. Then, the temperature was increased from 100 to 800 K and annealed to 300 K gradually leading to the desorption of the excess ligands away from the Ag nanoparticle surface. Finally, the whole system was allowed to equilibrate at 300 K for 1 ns with an integration time step of 1 fs. The Ag nanoparticle was kept frozen during the MD simulations to reduce computational cost.

## Additional information

**How to cite this article:** Chen, X. *et al.* Atomistic electrodynamics simulations of bare and ligand-coated nanoparticles in the quantum size regime. *Nat. Commun.* 6:8921 doi: 10.1038/ncomms9921 (2015).

## Supplementary Material

Supplementary Information Supplementary Figures 1-6, Supplementary Table 1, Supplementary Discussion, Supplementary Methods and Supplementary References.
