# Supplementary material for: Atomistic electrodynamics simulations of bare and ligand-coated nanoparticles in the quantum size regime
Source: Nat Commun. 2015 Nov 10;6:8921. doi: 10.1038/ncomms9921 (PMC5512832; doi:10.1038/ncomms9921)
Supplement: Supplementary Information — Supplementary Figures 1-6, Supplementary Table 1, Supplementary Discussion, Supplementary Methods and Supplementary References. [file ncomms9921-s1.pdf]

1 Supplementary Figures

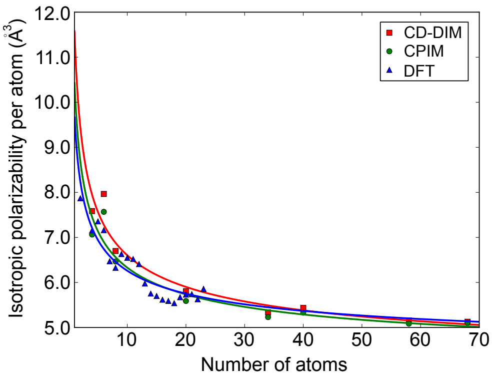

Supplementary Figure 1. Polarizability per atom of silver clusters as a function of the cluster size. The symbols represent the cd-DIM results (■), CPIM results (•)[1] and DFT results (▲) of Pereiro and Baldomir[2]. The solid line show the prediction from the jellium model.

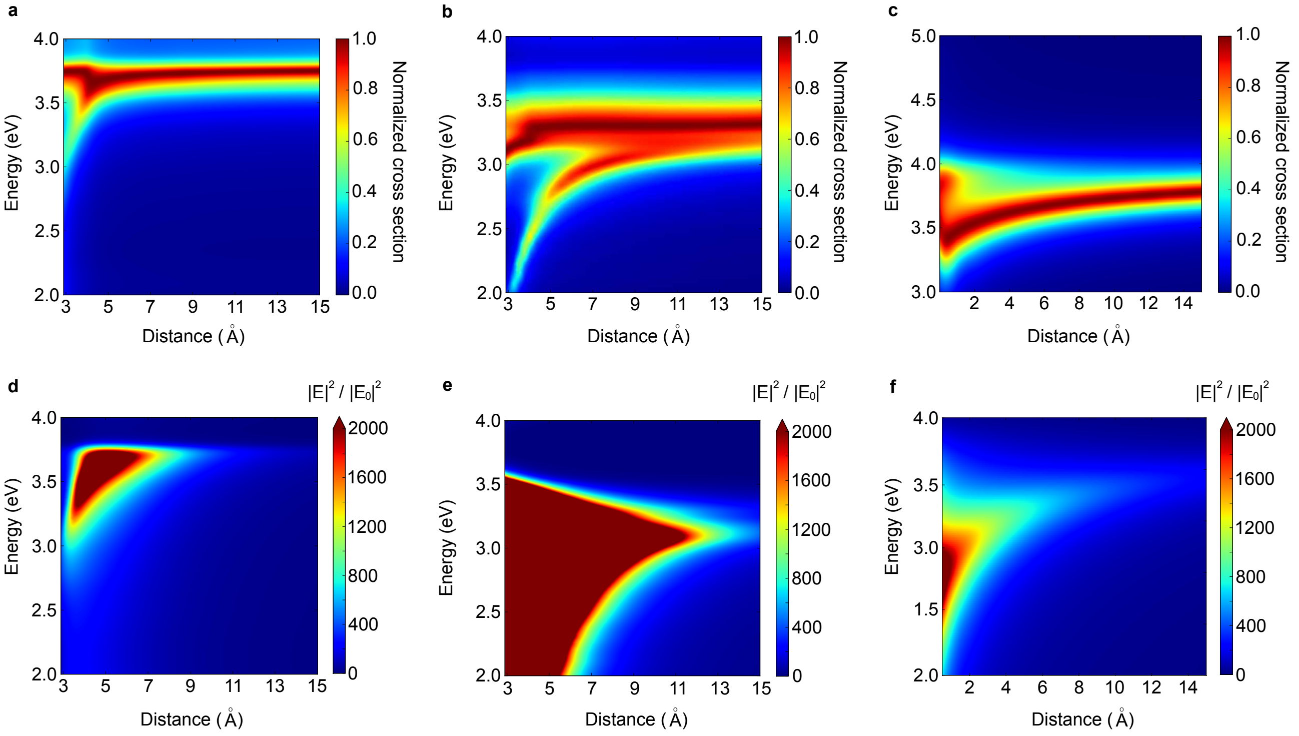

Supplementary Figure 2. Comparison of optical properties of the Ag nanoparticle dimers separated by various gap distances. The plasmonic response predicted by cd-DIM (a,d), the DDA model (b,e) and the GNOR mode (c,f). For cd-DIM and DDA two Ag icosahedral nanoparticles are aligned with the fivefold axes of rotation. The gap is defined as the length between two tip Ag atoms. For GNOR we used two nanorods with radius of 2.46 nm. The absorption spectrum and electric field enhancement at the center of gap distance as the function of the gap distance are illustrated in (a,b,c) and (d,e,f), respectively. The normalized absorption efficiency and the electric field E are considered at the dimer axis direction.

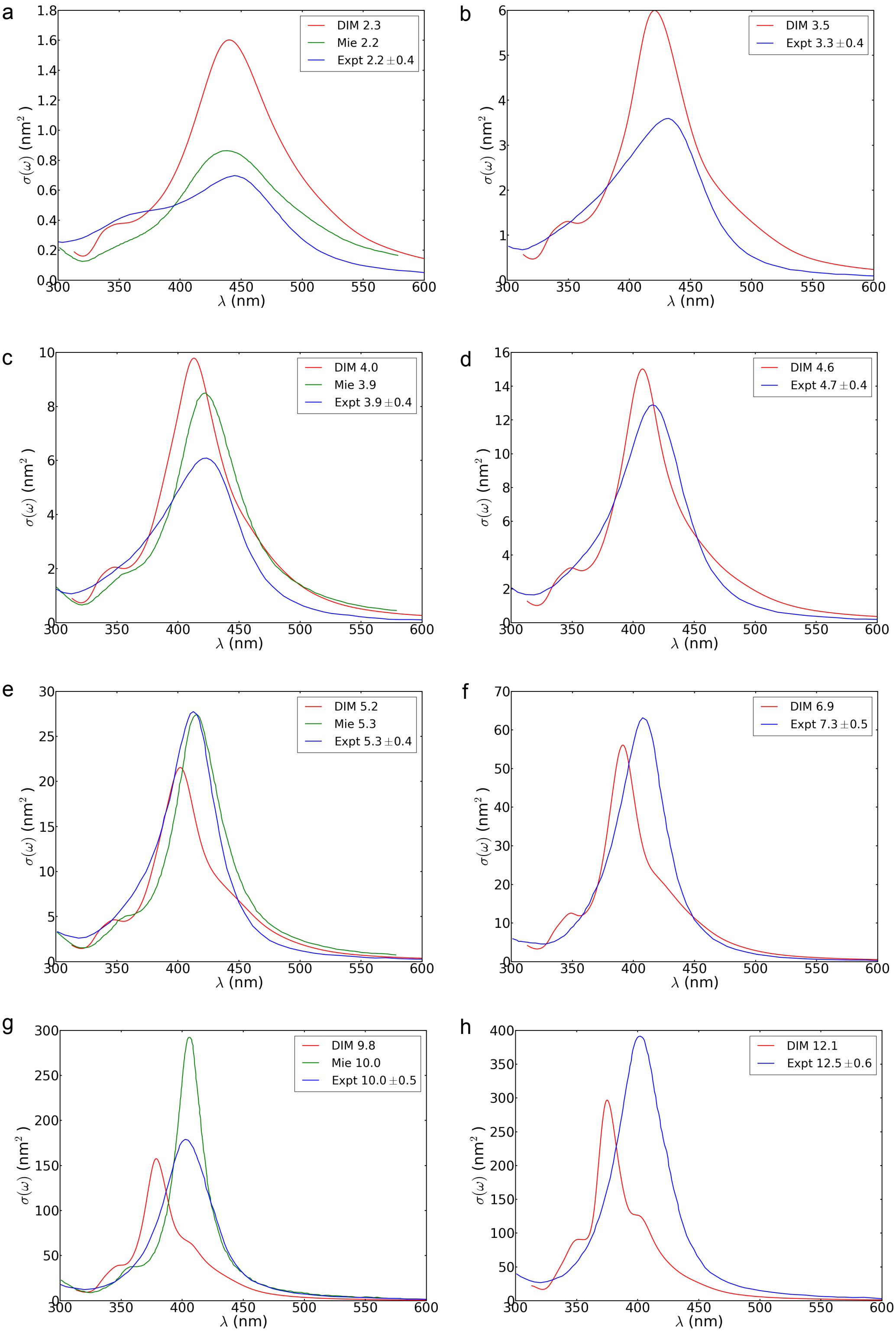

Supplementary Figure 3. Comparison of the plasmonic absorption spectra of the Ag nanoparticles obtained from cd-DIM (in red), multilayer Mie theory (in green)[3] and experiment (in blue)[3]. The nanoparticle diameter (in nm) shown in the figure's label.

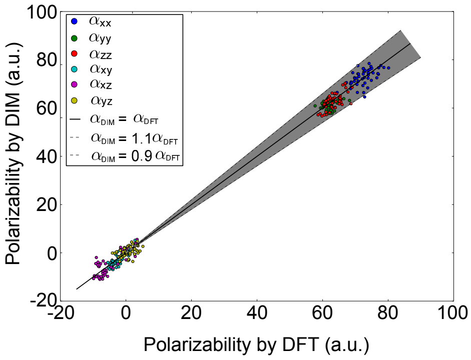

Supplementary Figure 4. Comparison of the polarizability calculated by DIM and DFT. The polarizability (solid circle) predicted by DIM show good agreement with the DFT result. The shaded region show deviation within ±10% from the DFT results.

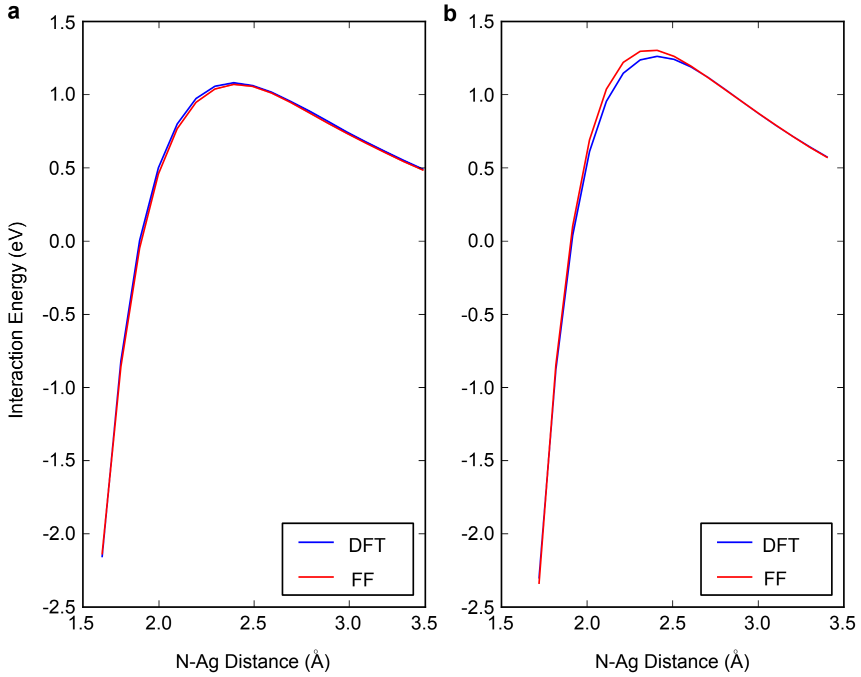

Supplementary Figure 5. Comparison of interaction energies calculated by DFT (in blue) and force field (in red) approaches with the various of N-Ag distances. The interaction energy as a function of N-Ag distance is responsible to the attraction or repulsion between Ag_{56} cluster and *n*-butylamine, which is perpendicular (a) or parallel (b) to the Ag(111) facet.

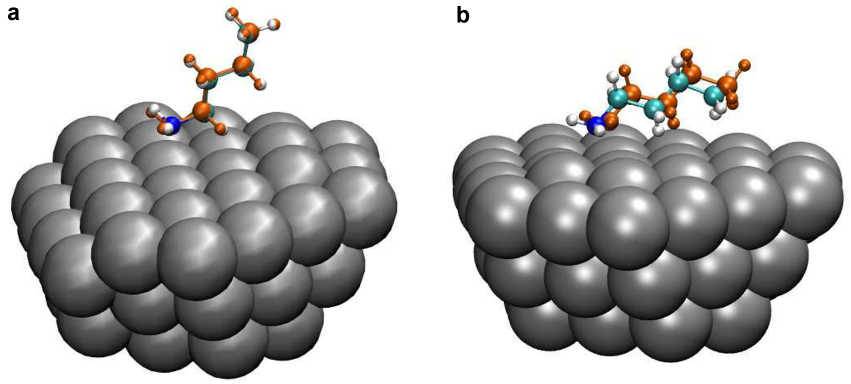

Supplementary Figure 6. Comparison of the optimized geometries of n-butylamine sitting on Ag_{56} cluster from DFT and force field (in orange) calculations. The *n*-butylamine adopts perpendicular (a) and parallel configurations (b) with respective to Ag(111) facet.

2 Supplementary Tables

Supplementary Table 1. Morse potential parameters and the scaling factor in electron-density function of EAM potential.

	D	α	r	f
Ag-C	0.000715	1.614	4.667	
Ag-N	0.1155	2.0599	2.5562	3.155
Ag-alkane H	0.000306	2.370	3.203	
Ag-alkane H[a]	0.000210	2.276	2.644	
Ag-amino H	0.000388	2.856	3.220	

[a] H is adjacent to N

3 Supplementary Discussion

Comparison with GNOR simulations

In Supplementary Fig. 2, we present a comparison between the optical properties of Ag nanoparticle dimers calculated using cd-DIM, DDA and GNOR. The cd-DIM and DDA results are for the nanoparticle dimers discussed in the main text. The GNOR result is based on a 2D model and thus represents infinite Ag nanorods. The GNOR simulations were done in the RF module of the COMSOL Multiphysics package (version 4.4) using the methods developed by Toscano *et al*[4]. Two 2.46 nm radius rods were placed 1 to 10 Å apart (in 1 Å step size). The magnitude of the electric field at a point equidistant between the nanorods was calculated as a result from an external field traveling along the long axis of the system, oscillating along the short axis.

Comparison with multilayer Mie simulations

In Supplementary Fig. 3, we present a comparison of the absorption cross section obtained using the multilayer Mie model, cd-DIM and experiment. The Mie results and experimental data are taken from Supplementary Reference 3. Overall, we find good agreement between the different methods. For the larger size we see that the position of resonance peak predicted using Mie theory is in better agreement with the experiments, but the intensity predicted by cd-DIM is in better agreement.

4 Supplementary Methods

Parametrization of classical force field

The general AMBER force field (GAFF)[5,6] was employed to describe the inter- and intramolecular interactions of the Oleylamine (OAm) ligands. However, there is a lack of the well parameterized force field for describing the Ag-OAm interactions. In order to describe the interactions between the ligand the nanoparticle, we modified a Ag-OAm embedded-atom-method (EAM) potential combined with a Morse potential for describing the Pauli repulsion and DFT-D2[7] dispersion corrections to the long-range van der Waals (vdW) attraction for chemical bonding. This approach has previously been used to simulate ligands binding to Ag surface[8,9]. The total energy of atom i is defined as

$$E_i = F_\alpha\left(\sum_{i\neq j}\rho_\beta(r_{ij})\right) + \frac{1}{2}\sum_{i\neq j}\phi_{\alpha\beta}(r_{ij}) \tag{1}$$

where F is the embedding energy which is a function of the atomic electron density ρ, and ϕ is the pair potential interaction between atoms i and j of element types α and β. To describe the chemical bond between Ag-N, we introduce an additional one-way electron-density function to the total electron density[8,9], which is represented as

$$\rho_\beta(r_{ij}) = \sum_{i\neq j}^{\mathrm{Ag}}\rho_{\mathrm{Ag-Ag}}(r_{ij}) + \sum_{i\neq j}^{\mathrm{N}}\rho_{\mathrm{N-Ag}}(r_{ij}) \tag{2}$$

It is assumed that $\rho_{\mathrm{N-Ag}}$ is proportional to $\rho_{\mathrm{Ag-Ag}}$, and, thus we adopt a scaling factor f to evaluate $\rho_{\mathrm{N-Ag}}$:

$$\sum_{i\neq j}^{\mathrm{N}}\rho_{\mathrm{N-Ag}}(r_{ij}) = f\rho_{\mathrm{Ag-Ag}}(r_{ij}) \tag{3}$$

where the pair potential is written in terms of Ag-Ag pair potential taken from the tabulated EAM potential of Ag (http://www.ctcms.nist.gov/potentials)[10]. The Morse potential is given by

$$\phi_{\text{Morse}}(r_{ij}) = D_{0,\text{Ag-L}}\left[e^{-2\alpha_{\text{Ag-L}}(r_{ij}-r_{0,\text{Ag-L}})} - 2e^{-\alpha_{\text{Ag-L}}(r_{ij}-r_{0,\text{Ag-L}})}\right] \tag{4}$$

where $D_{0,\text{Ag-L}}$, $\alpha_{\text{Ag-L}}$ and $r_{0,\text{Ag-L}}$ are the parameters of Morse potential describing the short-range interactions between Ag and OAm ligands. The long-range vdW interaction formulated in the DFT-D2 framework is given by[7]

$$\phi_{\text{DFT-D2}} = -s_6 f_{\text{damp}}(r_{ij})\frac{C_6^{ij}}{r_{ij}^6} \tag{5}$$

where s_6 is the global scaling parameter taken to be 1.05, and C_6^{ij} denotes the dispersion coefficient for atom pair ij, which is computed by means of the combination rule,

$$C_6^{ij} = \sqrt{C_6^{ii}C_6^{ij}} \tag{6}$$

In Eq. 5, $f_{\text{damp}}(r_{ij})$ denotes the Fermi-type damping function.

$$f_{damp}(r_{ij}) = \frac{1}{1+e^{-d(r_{ij}/R_0^{ij}-1)}} \tag{7}$$

Typically d=20 and R_0^{ij} denotes the sum of the vdW radii of atom pair ij.

The reference data used for the parametrization of the EAM-like potential were generated from DFT calculations, where we chose a model system consisting of n-butylamine (BAm) and a Ag_{56} cluster, see Supplementary Figure 6. We considered the perpendicular and parallel orientations of the BAm molecule with respect to the Ag(111) facet. For the DFT calculations we used the Becke-Perdew (BP86) XC-potential[12,13] with a triple-ζ polarized Slater type (TZP) basis sets to optimize the geometries using the ADF program package (http://www.scm.com)[14]. The 1s-3d core was frozen for Ag and scalar relativistic effects were taken into account by using the zeroth-order regular approximation (ZORA)[15–17]. Moreover, the dispersion energy was calculated by the DFT-D3 approach[18]. Only small differences in the geometry optimization were found by the DFT-D2 and DFT-D3 and thus the simpler D2 dispersion model was used in building the force field.

The parameters in the Morse potential and the scaling factor in the one-way electron-density function were found by minimizing the following cost function

$$\chi = \sum_{\text{perp,para}}\Bigg(\sqrt{\frac{\sum_{i,j,k}(f_{ijk}^{\text{FF}} - f_{ijk}^{\text{DFT}})^2}{\sum_{i,j,k} n_{ijk}}} + \sqrt{\frac{(E_i^{\text{inter,FF}} - E_i^{\text{inter,DFT}})^2}{\sum_i n_i}}\Bigg) \tag{8}$$

Here f_{ijk} represents the force along the Cartesian component k originating from the Ag cluster on atom j in the molecular configuration i. $\text{E}_i^{\text{inter}}$ denotes the interaction energy between BAm and Ag cluster in configuration i. The optimized parameters are collected in Supplementary Table 1. 16 grid points used in the parametrization were generated by perpendicularly translating the BAm molecule around the optimized geometry in step of 0.1Å with respective to the Ag(111) facet, while the geometries of BAm and Ag cluster are fixed.

A comparison of interaction energies obtained from DFT and the force field for BAm is represented in Supplementary Fig. 5 and the structure difference is illustrated in Supplementary Fig. 6. Overall, we see that the binding energy for the two orientations is correctly described using the force field. Comparing the geometries optimized by the force field and DFT for the perpendicular and parallel configurations, we find the RMSD are 0.042 Å and 0.439 Å, respectively.

Parameterization of atomic radii to describe the static polarizability

The atomic radii representing the surface and bulk atoms in cd-DIM were chosen to describe the mean static polarizability of small metal clusters as compared with DFT results. The size-dependence of the static polarizability can be described by a jellium model[1,19].

$$\overline{\alpha}_{\text{atom}} = \frac{(N^{1/3}r_{\text{WS}} + \delta)^3}{N} \tag{9}$$

where r_{WS} is the Wigner-Seitz radius of the bulk metal and δ represents the spillout of the electrons from the surface of a metallic sphere. In Supplementary Fig. 1 we present the data obtained using cd-DIM for Ag clusters

with $N \le 68$ that was previously used to study the polarizability using the capacitance-polarizability interaction model (CPIM)[1]. For comparison we have also included DFT data taken from Supplementary Reference 2. Fitting the cd-DIM results to the Jellium model gives $r_{\mathrm{WS}} \approx 1.54$ Å and $\delta \approx 0.72$ Å. Using the DFT data taken from Supplementary Reference 1, we find $r_{\mathrm{WS}} \approx 1.56$ Å and $\delta \approx 0.63$ Å, and using the DFT data from Supplementary Reference 2 gives $r_{\mathrm{WS}} \approx 1.63$ Å and $\delta \approx 0.45$ Å. These results are also in agreement with the values of $r_{\mathrm{WS}} \approx 1.58$ Å and $\delta \approx 0.74$ Å reported in Supplementary References 20, 21.

To obtain the atomic radii needed for describing the ligands we choose to minimize the difference between cd-DIM and DFT polarizability. The multiple configurations of BAm were selected from MD trajectories and used to calculate the polarizability using DFT. In the MD simulations, BAm was placed in the center of 15×15×15 Å^3 periodic box, and the linear and angular momenta were zeroed. Followed by energy minimization, the temperature was increased gradually from 0 to 300 K under the NVT ensemble within 50 ps, and subsequently the whole system was equilibrated for 10 ns with an integration time step of 0.5 fs. Periodic boundary conditions with a cutoff distance of 12 Å for vdW and electrostatic interactions were employed. The static polarizability was calculated by DFT at the B3LYP/aug-cc-pVDZ level of theory using the program package NWChem 6.1.1[22].

The atomic radii of BAm were then determined by minimize the following cost function

$$\chi = \sqrt{\frac{\sum_{i,\alpha,\beta}(\alpha^{\mathrm{DIM}}_{i,\alpha\beta} - \alpha^{\mathrm{DFT}}_{i\alpha\beta})^2}{3N}} \tag{10}$$

where $\alpha_{i,\alpha\beta}$ represents the each component in the polarizability tensor of configuration i, and N is the number of configurations, which is set to 50. The comparison between the polarizability calculated by DIM and DFT is shown in Supplementary Fig. 4.

Supplementary References

[1] Jensen, L. L. & Jensen, L. Electrostatic interaction model for the calculation of the polarizability of large noble metal nanoclusters. *J. Phys. Chem. C* **112**, 15697–15703 (2008).

[2] Pereiro, M. & Baldomir, D. Structure of small silver clusters and static response to an external electric field. *Phys. Rev. A* **75**, 033202 (2007).

[3] Peng, S., McMahon, J. M., Schatz, G. C., Gray, S. K. & Sun, Y. Reversing the size-dependence of surface plasmon resonances. *Proc. Natl. Acad. Sci. USA* **107**, 14530–14534 (2010).

[4] Toscano, G. *et al.* Nonlocal response in plasmonic waveguiding with extreme light confinement. *Nanophotonics* **2**, 161–166 (2013).

[5] Wang, J., Wolf, R. M., Caldwell, J. W., Kollman, P. A. & Case, D. A. Development and testing of a general amber force field. *J. Comput. Chem.* **25**, 1157–1174 (2004).

[6] Wang, J., Wang, W., Kollman, P. A. & Case, D. A. Automatic atom type and bond type perception in molecular mechanical calculations. *J. Mol. Graph. Mod.* **25**, 247–260 (2006).

[7] Grimme, S. Semiempirical gga-type density functional constructed with a long-range dispersion correction. *J. Comput. Chem.* **27**, 1787 (2006).

[8] Grochola, G., Snook, I. K. & Russo, S. P. Computational modeling of nanorod growth. *J. Chem. Phys.* **127**, 194707 (2007).

[9] Zhou, Y., Saidi, W. A. & Fichthorn, K. A. A force field for describing the polyvinylpyrrolidone-mediated solution-phase synthesis of shape-selective Ag nanoparticles. *J. Phys. Chem. C* **118**, 3366–3374 (2014).

[10] Williams, P. L., Mishin, Y. & Hamilton, J. C. An embedded-atom potential for the cu-ag system. *Modell. Simul. Mater. Sci. Eng.* **14**, 817 (2006).

[11] Perdew, J. P. Density-functional approximation for the correlation energy of the inhomogeneous electron gas. *Phys. Rev. B* **33**, 8822–8824 (1986).

[12] Becke, A. D. Density-functional exchange-energy approximation with correct asymtotic-behavior. *Phys. Rev. A* **38**, 3098–3100 (1988).

[13] Baerends, E. *et al.* Amsterdam density functional (2013).

[14] van Lenthe, E., Baerends, E. J. & Snijders, J. G. Relativistic regular two-component hamiltonians. *J. Chem. Phys.* **99**, 4597–4610 (1993).

[15] van Lenthe, E., Baerends, E. J. & Snijders, J. G. Relativistic total energy using regular approximations. *J. Chem. Phys.* **101**, 9783–9792 (1994).

[16] van Lenthe, E., Ehlers, A. & Baerends, E.-J. Geometry optimizations in the zero order regular approximation for relativistic effects. *J. Chem. Phys.* **110**, 8943–8953 (1999).

[17] Grimme, S., Antony, J., Ehrlich, S. & Krieg, H. A consistent and accurate ab initio parametrization of density functional dispersion correction (dft-d) for the 94 elements h-pu. *J. Chem. Phys.* **132**, 154104 (2010).

[18] Snider, D. R. & Sorbello, R. S. Density-functional calculation of the static electronic polarizability of a small metal sphere. *Phys. Rev. B* **28**, 5702–5710 (1983).

[19] de Heer, W. A. The physics of simple metal clusters: experimental aspects and simple models. *Rev. Mod. Phys.* **65**, 611 (1993).

[20] Bennett, L. H., Mebs, R. W. & Watson, R. E. Solute Knight Shifts in Noble Metals. *Phys. Rev.* **171**, 611–626 (1968).

[21] Valiev, M. *et al.* Nwchem: A comprehensive and scalable open-source solution for large scale molecular simulations. *Comput. Phys. Commun.* **181**, 1477–1489 (2010).
